# Supplementary material for: Antibody-recruiting molecules based on mimotope fusion peptides and a dimeric antibiotic
Source: bioRxiv. 2026 Sep 30:2026.09.29.755485. Preprint. [Version 1] doi: 10.64898/2026.09.29.755485 (PMC13648653; doi:10.64898/2026.09.29.755485)
Supplement: Supplementary file 1 [file NIHPP2026.09.29.755485V1-supplement-1.pdf]

Fig. 1S

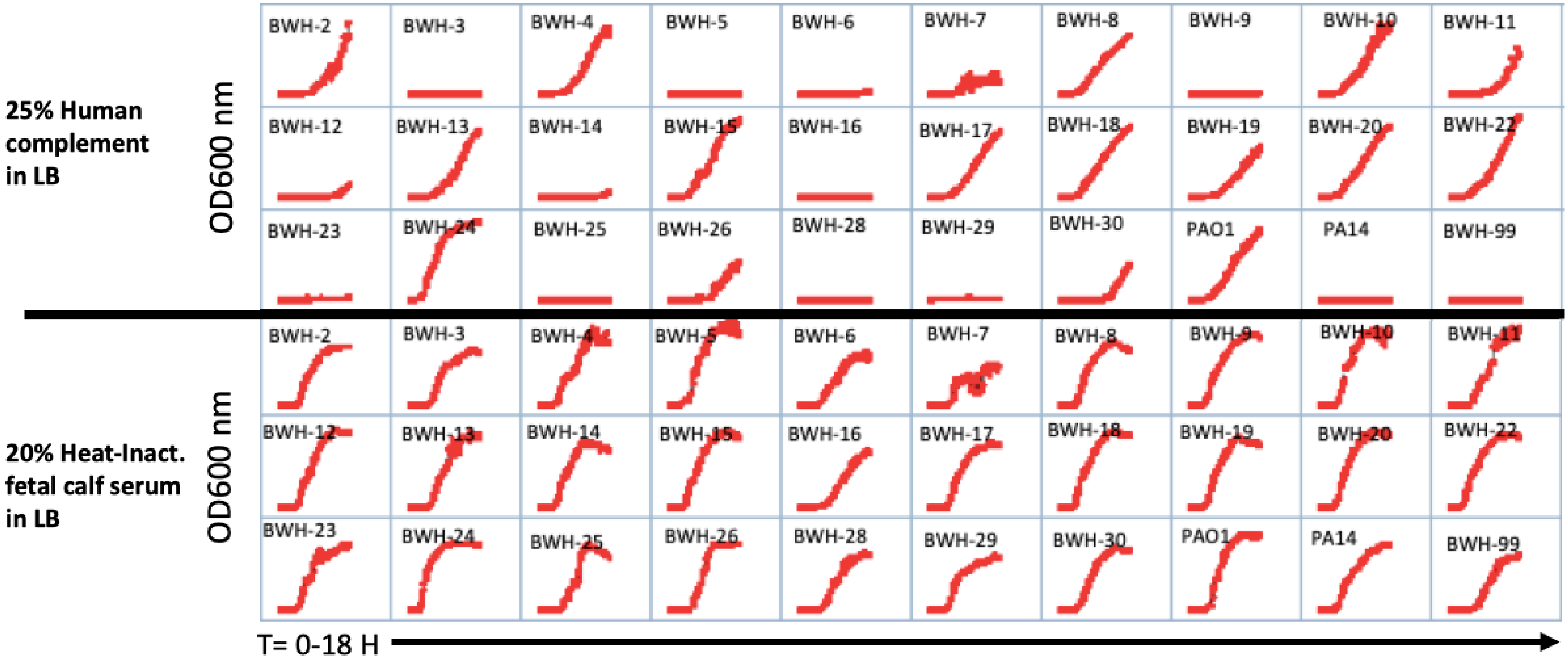
25% Human complement in LB
20% Heat-Inact. fetal calf serum in LB
OD600 nm
OD600 nm
BWH-2
BWH-3
BWH-4
BWH-5
BWH-6
BWH-7
BWH-8
BWH-9
BWH-10
BWH-11
BWH-12
BWH-13
BWH-14
BWH-15
BWH-16
BWH-17
BWH-18
BWH-19
BWH-20
BWH-22
BWH-23
BWH-24
BWH-25
BWH-26
BWH-28
BWH-29
BWH-30
PAO1
PA14
BWH-99
T= 0-18 H
